# Preventing slips, overruns, and cancellations: Application of system accident investigations and theory to the understanding and prevention of engineering project failures

**DOI:** 10.1371/journal.pone.0229825

**Published:** 2020-03-06

**Authors:** Diane C. Aloisio, Karen Marais

**Affiliations:** Department of Aeronautics and Astronautics, Purdue University, West Lafayette, Indiana, United States of America; Indian Institute of Technology Madras, INDIA

## Abstract

Organizations that develop or operate complex engineering systems are plagued by systems engineering failures like schedule overruns, budget exceedances, and project cancellations. Unfortunately, there is not much actionable guidance on why these failures happen or how to prevent them. Our approach contains two novel aspects. First, we argue that system accidents and other failures in systems engineering are manifestations of similar underlying problems. Therefore, we can leverage the literature on accident causation and the many publicly available accident investigation reports to better understand how and why failures in systems engineering occur, and to identify ways of preventing them. Second, to address the lack of concrete guidance on identifying and preventing incipient failures, we provide specific examples of each type of failure cause and of the recommendations for preventing these causes. We analyzed a set of 30 accidents and 33 project failures, spanning a range of industries, and found 23 different failure causes, most of which appear in both accidents and other project failures, suggesting that accidents and project failures do happen in similar was. We also identified 16 different recommended remedial actions. We link these causes and recommendations in a cause-recommendation network, and associate over 900 specific examples of how these causes manifested in failures, and over 600 specific examples of the associated recommended remedial actions, with each cause or recommendation.

## Introduction

Few engineering projects are completed on-time, within proposed budget, and with the negotiated features and functions. In 2008, only eleven of 72 major United States defense programs were on schedule, on budget, and met performance criteria [1]. Since then, U.S. aerospace and defense programs have only worsened: total cost overruns “have risen from 28 percent to 48 percent, from 2007 through 2015” [2]. The U.S. Government Accountability Office (GAO) suggested that many current programs are vulnerable to “cost growth or schedule delays” [3]. The consumer goods sector too suffers from these problems, as shown by the Xbox 360 “Red Rings of Death” [4] and Ford Explorer rollover problems [5].

Previous studies have identified problems in systems engineering and project management, but neither the research community nor practitioners have been able to identify fully or prevent the causes of project failures. Some project failure studies provide in-depth analysis on a few large-scale failures. Keil & Mähring [6] performed an in-depth study of two IT project failures: the Eurobank deposit system and the California DMV database. They analyzed these failures using an “escalation” framework, which describes three distinct phases project failures experience: drifting, treating symptoms, and rationalizing continuation.

Other project failure studies look at patterns across a moderate number of large-scale failures. Shore [7] studied a variety of project failures from different industries and identified “systematic biases” throughout these cases, such as “overconfidence” and “conservatism”. Williams et al. [8] studied eight cases of how project assessments identified early warning signs of impending project failure. They provide descriptive cases of warning signs at various stages in the project lifecycle (e.g. “lack of a culture of openness and good communication between actors” during the early stages of a project) from a project management perspective. Nutt [9] identifies poor decisions in a wide range of industries in 15 detailed case studies. For example, he describes how “ambiguous directions” led Disney to build EuroDisney to realize “Walt’s Dream”, rather than basing its decisions on market demand and profitability. Newman [10] analyzed 50 space failures from a high-level systems engineering perspective and discussed broad categories into which many of the causes of these failures fell (e.g., Design, Manufacturing, or Human Error).

Lastly, some project failure studies gather data on a sufficiently large number of smaller-scale failures or “incidents” to perform a statistical analysis. Konstandinidou et al. [11] studied over 1,000 incidents in the Greek petrochemical industry and identified causal factors, such as “inadequate procedures” and “lack of communication” that contributed to these incidents. They found that causal factors such as human factors (e.g. “errors of omission”) contributed more to injury and workplace absences, and organizational factors (e.g. “inadequate procedures”, “inadequate training”) contributed more to material damage. These types of analyses aim to achieve formal statistical significance or at least strength in numbers, but may sacrifice the depth of the case study approach.

Depending on the study type, guidance on project failures is either highly contextualized or very general. In-depth analyses tend to focus on highly specific criticism that are difficult to generalize to other situations. At the other extreme for studies that analyze large number of failures, guidance on preventing failures is often quite general, such as “put your best people on the project and resolve the root causes” [6] or “top management needs to provide unambiguous reinforcing messages from time to time” [12]. Such guidance is certainly valid (clearly one would not want to put one’s worst people on an important project!), but it also tends to only address problems at the surface and not the underlying reasons for these problems (Why weren’t the best people on the project? And where should the not-best people be placed?).

It is generally difficult to obtain detailed information about project failures. Most organizations do not readily release specific details on what lead to internal problems, and investigations are usually not overseen by a neutral auditing organization. For a system accident, however, information on what lead to the failure is usually much more widely available and experts in accident causation usually provide specific guidance on how to fix the underlying problems in the organization.

In this paper, we compare in-depth examples from reports on accidents and project failures and develop a coding scheme to help us identify patterns across these failures. We also use the lessons learned from accidents to help identify specific, pointed preventive measures for project failures. We use our findings to develop a cause-recommendation network that shows how causes tend to cluster, which recommendations are appropriate for which causes, and how the causes and recommendations are manifested in a range of industries.

We begin with a brief review of the state-of-the-art research in accident causation and how it can be applied to project failures. Next, we describe our case selection dataset and how we extracted and analyzed findings and recommendations from failure reports. We then build networks of causes in accidents and project failures as well as a network of causes linked to recommendations, and illustrate potential applications for this cause-recommendation network. We conclude the paper with ideas for future work.

A note on definitions: Both system accidents and project failures are “undesired and unplanned (but not necessarily unexpected) event[s] that result in (at least) a specified level of loss” [13]. We use “system accident” (which we shorten to “accident” for ease of reading) to refer to those events that directly result in loss of life, injury, or damage to property [14]. System accidents are a generalization of “process accidents” in the chemical process industry (cf. [15]). We do not consider here occupational safety accidents such as falls from ladders or mishandling of lathes. We use “project failures” for all other undesired project events, such as failure to achieve mission objectives, budget or schedule overruns, cancellations, and quality or performance issues [14]. In both cases, this paper focuses on systems that are technologically and organizationally complex, and usually expensive, both in terms of direct and indirect losses.

## What have we learned from accident research?

A range of accident modeling techniques that help explain how accidents are caused is available. Accident investigation reports, and subsequent meta-analyses of these reports, have revealed that accidents across industries have similar causes despite occurring in different scenarios. This section provides a brief review of the literature; for a more extensive discussion see Saleh et al. [16].

Theories and models on accident causation have become increasingly sophisticated, beginning with considering accidents as simple chains of human errors and physical failures. Our current understanding is that accidents result from a complex web of interactions, many of which are, or at least appear to be, locally and temporally rational. Man-made disasters theory is an early and influential articulation of this perspective [17]. It posits that accidents are not the result of chance events, but rather occur as a result of a build-up of errors and hazards over time. Man-made disasters theory helps explain why accidents occur even at organizations that have safety programs in place and claim to value safety. When members of the organization collectively follow the safety rules and procedures less well and less frequently or commit other mundane day-to-day errors, accidents may arise.

Human factors (ergonomics) and organizational factors studies have provided understanding of why people make errors. For example, people routinely violate procedures—because doing so often allows them to perform tasks more quickly and efficiently, sometimes at the cost of safety. James Reason’s work, of which the Swiss cheese model is one of the best-known aspects, is an influential successor to Turner’s work [18]. The Swiss cheese model views safety as being maintained by layers of defense, which develop and close holes over time as for example procedure compliance decreases and increases. When there are sufficient holes, or when holes remain in place for long enough, accidents can shoot through the layers of defense. Reason also posited that accidents can be traced back to problems on four levels: specific acts, preconditions, supervision, and organizational influences. Each higher level drives the problems below it. Based on these layers, Shappell and Wiegmann [19] developed a taxonomy of accident causes and codified them in the Human Factors Analysis and Classification System (HFACS).

The view that system safety is a control problem that requires a systems perspective has emerged as the current leading theory. The control-theoretic perspective on system safety grew out of general systems theory and sees accidents as resulting from the absence or breach of defenses, be they technical or organizational, or from the violation of technical or organizational safety constraints [20] [21] [22] [16]. Absences and breaches of defenses and safety constraint violations can occur at any level of an organization.

Progress in accident theory and modelling is both informed by and drives the growing recognition that accidents, though often differing in their details, share root causes, whether expressed as lurking pathogens in Swiss Cheese, layers or types of errors in HFACS, or control flaws in Rasmussen or Leveson’s work [e.g., [23] [21] [10]]. For example, the technicians working on the NOAA N-Prime Satellite committed a skill-based memory lapse error when they failed to notice that bolts holding the spacecraft to a working surface were missing, despite wiping the surface and not detecting interference from the bolts, resulting in the spacecraft toppling when they attempted to move the working surface [24]. After a Boeing 747 operated by China Airlines experienced a tailstrike incident, personnel committed a rule-based mistake when they did not follow maintenance procedures requiring them to remove the entire potentially damaged portion of the tail. The material eventually fatigued to the point of failure on flight 611 [25]. Both of these failures had problems with their organizational climates and communication: the NOAA N-Prime crew had an atypical mix of authority on the morning of the incident, which was not conducive to open discussion and shared responsibility, and the Boeing repair procedures and customer communications channels did not instruct the China Airlines crew on how to perform tailstrike repair correctly.

Here, then, we posit that, just as accidents share many causes, project failures share causes with accidents in particular, and also with other project failures. We explore this idea in the next section.

## Method

This section describes the dataset, the resulting set of accident and project failures causes, and the linked set of recommendations for preventing these failures. Our resulting data is hosted on the Purdue University Research Repository [26].

### Dataset description

There are few detailed publicly-available reports on project failures. We identified 33 cases with systems engineering-related causes, with sufficient detail, that span a range of industries, and that occurred relatively recently (from 1979 to 2015). We also selected novel projects that involved state-of-the-art, advanced technology (e.g. Mars Polar Lander), as well as ongoing projects that make improvements to existing designs (e.g. Boeing 787 Dreamliner). In contrast, no industry is free of publicly available accident investigation reports, with the United States National Transportation Safety Database, and Chemical Safety Board being two examples of readily available accident report sources. We selected 30 accidents spanning a wide range of industries. For more information on the types of sources we used and the implications of those sources, see [27]. Table 1 shows our cases.

**Table 1:** Accidents and project failures [27].

Failure Category	Industry	Case
**Project Failure**	**Consumer product**	Apple Newton MesssagePad, HD DVD, Google Glass, Zune, Windows Vista, Xbox 360, Ford Explorer, Merck Vioxx Drug, Boeing 787 Dreamliner, Iridium Satellite Phone, Segway
	**Infrastructure project**	FAA STARS, Maritime Automated Identification system, California DMV, Healthcare.gov, Denver Airport Baggage system, Boston Big Dig
	**Government acquisition**	Seawolf Navy Submarine, Future Combat Systems, AMRAAM, Littoral Navy Ship, DEA Plane, F-35 Lightning II, F-22 Raptor, V-22 Osprey, Future Imagery Architecture
	**Space Mission**	X-33 VentureStar, Mars Climate Orbiter, NOAA N-Prime, SOHO Loss, Mars Polar Lander, Titan Mission Failure, Hubble
**Accident**	**Aerospace**	Challenger, Columbia, TWA 800, Alaska 261, Colgan 3407, Aloha 243, ValuJet 592, China 611, Swissair 111
	**Energy**	B.P. Texas City Refinery, Kleen Energy, Xcel Energy Hydro Plant, Imperial Sugar, Chernobyl, Fukushima Nuclear, Exxon Valdez, Deepwater Horizon, Three Mile Island, Piper Alpha, Westray Mine, Buncefield Oil Storage, Pike River Mine, Upper Big Branch Mine, Cyanide spill, Bhopal
	**Infrastructure**	Walkerton, North Battleford, New Orleans Levee, Channel Tunnel, Lac-Mégantic Train

### Cause extraction

Our approach consists of five steps: (1) identifying findings in reports, (2) seeding our coding process with summary statements for findings from a subset of our cases, (3) applying the findings to a modified STAMP model to identify where in the design process they fall, (4) iteratively developing a coding scheme for the findings, and (5) coding the remaining findings to remove extraneous detail. We illustrate this process with the Deepwater Horizon oil spill and the F-35 Lightning II schedule and budget exceedances.

We began by extracting findings on the Deepwater Horizon oil spill from the two available accident reports [28] [29]. Table 2 shows a subset of the 25 findings for the Deepwater Horizon oil spill. We extracted the findings of the F-35 Lightning II budget and schedule exceedances from four newspaper articles and a U.S. Department of Defense report [30] [31] [32] [33]. Table 3 shows a subset of the 25 finding extracts for the F-35 Lightning II.

**Table 2:** Deepwater horizon accident example statements and sources [27].

#	Report Extract (Finding)	Finding Summaries
1	“The crew could not perform the negative-pressure test using the drill pipe; it would open the top of the drill pipe on the rig, bleed the drill pipe pressure to zero, and then watch for flow. […] the crew tried to bleed the pressure down to zero, but could not get it below 266 psi. […] [The site leader] then insisted on running a second negative-pressure test, this time monitoring pressure and flow on the kill line rather than the drill pipe. […] [The crew] made a key error and mistakenly concluded the second negative test procedure had confirmed the well’s integrity.” [28, pp. 107–109]	Personnel inadequately addressed questionable test results.
2	“A blowout preventer can act as a barrier only if it is closed manually by the drilling crew or automatically as a result of a catastrophic event, such as a fire and explosion, which can trigger emergency backup systems. In manual operations, successful closure of the blowout preventer depends on several human decisions that must be made before a well kick can develop into a blowout. Otherwise, well pressures and well flow can exceed the design capabilities of the blowout preventer elements, leaving them unable to prevent or stop an active blowout.” [29, p. 14]	Designers underestimated the severity of well blowout
3a	“The crew may have been distracted by other matters.” [28, p. 111]	Operations management gave operators too many tasks to perform at once.
3b	“As the crew conducted the test, the drill shack grew crowded. The night crew began arriving to relieve the day shift, and Harrell brought the VIPs through as part of their tour.” [28, p. 5]	Operations management gave operators too many tasks to perform at once.
4	“The laboratory personnel conducted several tests, including a foam stability test, starting an approximately April 13. The first test Halliburton conducted showed once again that the cement slurry would be unstable. The Commission does not believe that Halliburton ever reported this information to B.P.” [28, p. 101]	Laboratory personnel did not alert management about poor test results.
5	“The [regulatory] agency’s management shortcomings were underscored, and compounded, by lack of communication and inconsistencies among its three regional offices for the Gulf of Mexico, the Pacific, and Alaska. […] by acting in parallel fashion, with little coordination in decision-making and resource allocation, program implementation, regulatory interpretation, and enforcement policies became inconsistent, undermining the integrity of MMS’s work.” [28, p. 78]	Regulatory body provided poor regulatory supervision of rig operations.
6	The regulator “does not require industry to identify and manage all safety critical elements and tasks through defined performance standards, nor does it require assurance and verification activities to ensure a safety critical element is appropriate, available, and effective throughout its life cycle. [29, p. 16]	Regulatory body provided poor regulatory supervision of rig operations.

**Table 3:** F-35 Project failure example statements and sources.

#	Report Extract (Finding)	Finding Summary
1	“Our assessment documented several findings citing inadequacies in Lockheed Martin’s oversight of its suppliers and management of subcontractor deliverables.” [32]	Development management poorly supervised suppliers.
2a	“Technological innovation, including heavy reliance on computer simulation, which could take the place of real-world testing, would keep costs down. […] Building an airplane while it is still being designed and tested is referred to as concurrency. In effect, concurrency creates an expensive and frustrating non-decision loop: build a plane, fly a plane, find a flaw, design a fix, retrofit the plane, rinse, repeat.” [31]	Computer simulations were inadequate tests to identify design problems.
2b	“Pentagon officials accepted Lockheed’s claim that computer simulations would be able to identify design problems, minimizing the need to make changes once the plane actually took to the sky. […] [But] early tests uncovered flaws unnoticed by the computer simulations.” [30]	Computer simulations were inadequate tests to identify design problems.
3a	“The Air Force, Marines and Navy all sought additional modifications to meet their needs, reducing commonality among the three models. A bigger problem was the fundamental concept of building one plane, with stealth technology, that could fly as far and fast as the Air Force wanted while also being able to land on the Navy’s carriers and take off vertically from Marine amphibious assault ships.” [30]	Development management tried to please too many customers in one limited design.
3b	“From the outset, critics have worried that by trying to meet so many missions for so many masters, the Joint Strike Fighter would end up being […] a ‘jack of all trades, and master of none.’ Take the matter of stealth technology, which helps an airplane elude detection. […] it doesn’t serve much purpose in a Marine Corps environment.” [31]	Development management tried to please too many customers in one limited design.
4	“We identified that Lockheed Martin did not maintain mission systems requirements traceability to the software-level requirements. […] Untraceable requirements cannot be verified for impact on system performance.” [32]	Development management did not keep track of requirements.

**Fig 1:**
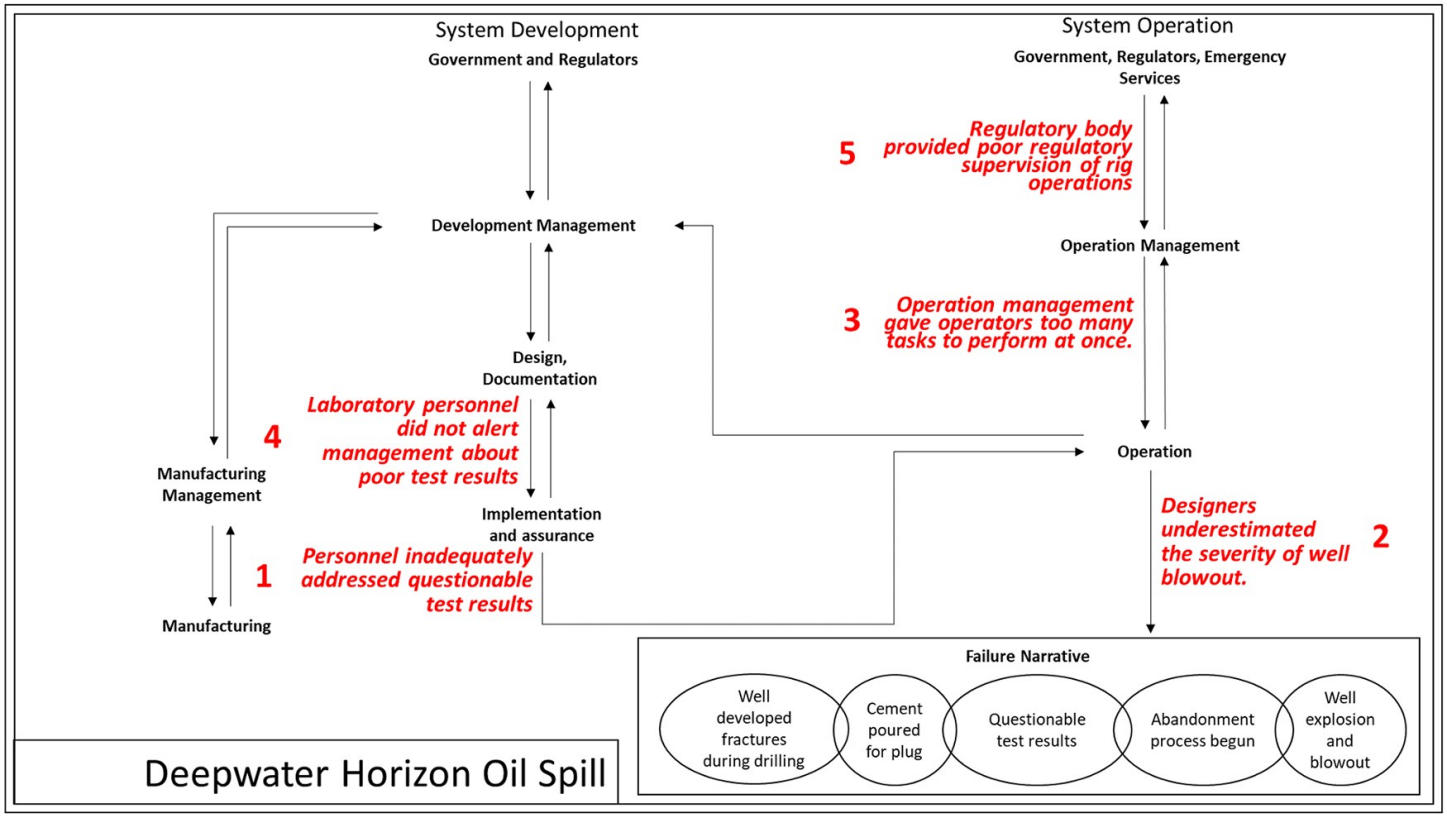
Deepwater Horizon causes applied to modified STAMP model.

Second, we modified the STAMP model [22] to help us systematically identify where and when in the design process the finding occurred and used it as a framework for classifying the findings by organizational level. Fig 1 shows the model for the Deepwater Horizon case with the finding summaries 1 through 5 from Table 2 placed at appropriate locations on the model. For more information on how we modified and used the STAMP model, see [34].

Next, we reworded each finding to retain the defining information but discard extraneous details. For example, we reworded the first finding in Table 2 to discard “crew” and “site leader” and replaced them with the more general “personnel”. The test the crew did indicated a potential problem, so they retested in a different way, rather than figuring out why they got unfavorable results in the first place. We summarized this finding as “insufficiently addressed questionable test results”. Many reports refer to the same instance of a particular problem more than once—for example in a body chapter and also in the conclusion. Cases with more than one report (e.g., Deepwater Horizon) also resulted in more than one extract referring to the same instance of a particular problem, as indicated for example in rows 5 and 6 of Table 2. Reports may also refer to different instances of the same problem, as indicated for example in rows 3a and 3b of Table 2. In Table 2, rows 5 and 6 discuss two different regulator shortcomings. We therefore counted these excerpts as two findings. In contrast, rows 3a and 3b both refer to the same instance of the same problem—accordingly we counted these excerpts as one finding.

The reports vary in how they specify the parties involved in a particular finding. Some reports contain extensive details, including names and roles (e.g., the Walkerton water contamination accident names particular people [35]). Some reports specify only the roles (e.g., the NTSB discusses the causes in terms of “pilot” or “co-pilot”). Johnson [36] describes the ambiguity that many accident reports contain because they use inconsistent natural language. When reports did not specify names or roles, we inferred the roles. For example, consider the third finding in Table 2, in which the oil rig crew was distracted by a VIP tour while conducting an important test in a small control room. We inferred from the report that the persons responsible for bringing the VIPs were in an operations management role.

Some investigation bodies record accidents using a coding system, such as the NTSB’s method for investigating aviation accidents [37]. This type of system allows the investigators to have a baseline from which to analyze multiple accidents at once. The NTSB coding system facilitates analysis of overall trends in accident causation. Here, we coded each statement into an “actor-causal action-object” structure, where the actor is the person (or group of people), the causal action is what they did, and the object provides detail about what the causal action was applied to. This coding structure allows us to compare failures with a baseline, like the NTSB’s scheme. The “object” acts like a modifier to a “causal action” and makes it specific to a failure type. For more examples of how this coding scheme can be applied to different causes, refer to [38].

**Fig 2:**
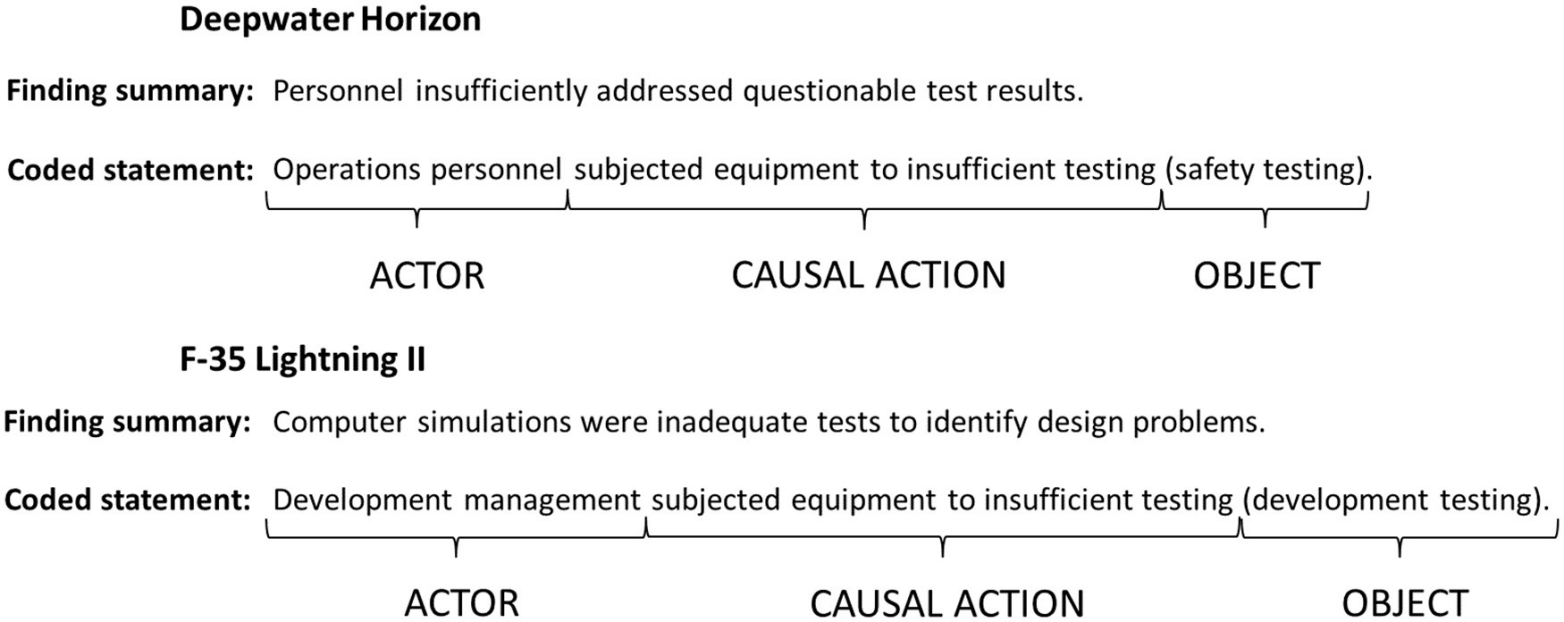
Actor-causal action-object structure for findings in different failures.

Fig 2 shows an example of two similar findings, from the Deepwater Horizon accident and the F-35 project failure. In both cases, testing was inadequate in some way, so we created a “subjected equipment to inadequate testing” causal action. In the Deepwater Horizon case, it was the personnel conducting the test who did not adequately investigate the questionable test results. Had they done so, they would likely have realized that they needed to redo the test. In contrast, on the F-35, development managers requested a form of testing (computer simulation) that was insufficient. Thus, we assigned responsibility to the development managers, rather than to the engineers conducting the simulations. The objects for each statement, “safety testing” and “development testing”, identify the specific type of testing.

**Fig 3:**
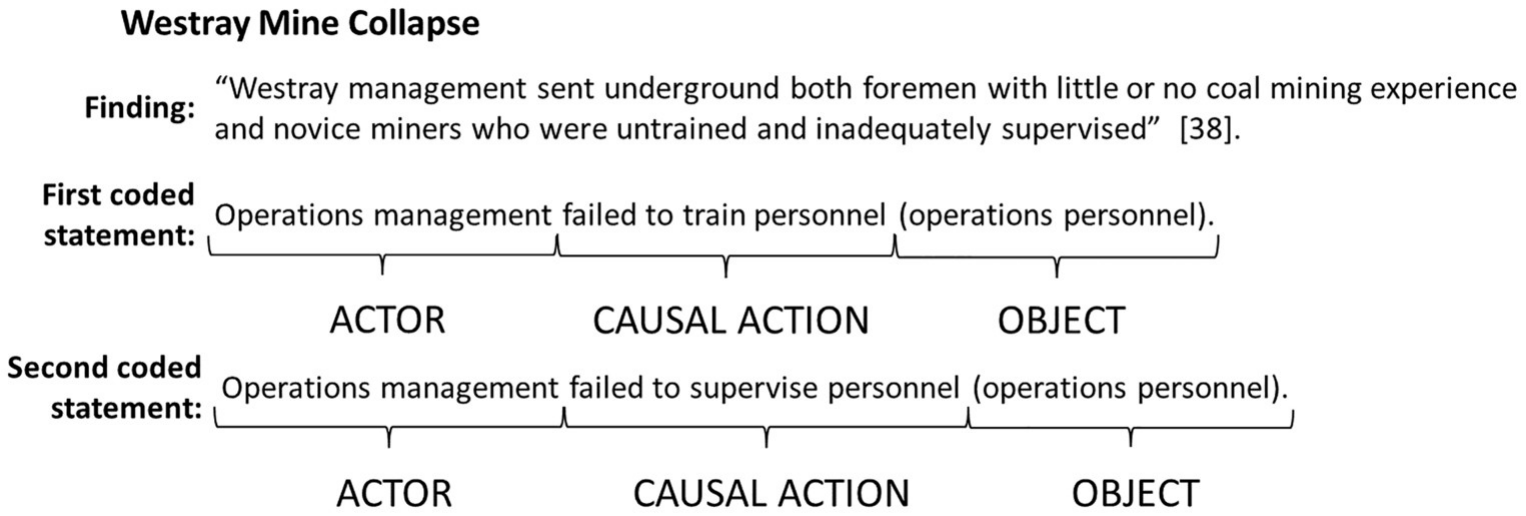
Actor-causal action-object structure for findings with multiple coded statements [39].

When a particular finding involved more than one actor, causal action, or object, we assigned additional unique actor-causal action-object codes to the finding to illustrate all facets of the finding. Fig 3 shows an example of a finding from the Westray Mine collapse to which we assigned two coded statements.

We identified a total of 966 findings, which we represent using a set of 23 causal actions, 9 actors, and 119 objects. Each causal action is associated with at least one object; for instance, “subjected to inadequate testing” has objects describing five types of testing: acceptance, development, quality, reliability, and safety testing. Other causal actions have more abstract objects. For example, “used inadequate justification”, has objects like “acquisition” and “hiring”.

We focus on the causal actions, as listed in Table 4. In the failures we studied, we found that different actors made similar mistakes (e.g., people at all levels of an organization keep poor records). People also made similar mistakes on different “objects” (e.g., poor records of different processes). Focusing on the “causal action” helps in identifying what went wrong rather than who to blame. In the remainder of this paper, we will simply refer to “causal actions” as “causes”.

**Table 4:** Cause definitions and presences [14].

Cause	Definition	Project Failure %	Accident %
Failed to supervise	Actor(s) in the organization failed to supervise people or a process properly.	76%	77%
Failed to provide resources	Actor(s) in the organization failed to provide adequate resources to a department; for instance, maintenance, marketing, or safety.	45%	47%
Failed to consider design aspect	Actor(s) in the organization failed to consider an aspect in the system design. In many cases, this causal action describes a design flaw, such as a single-point failure or component compatibility.	85%	83%
Lost tacit knowledge when employee departed	Personnel quit, were moved to a different project, or retired, and the organization failed to sustain the knowledge base without these persons.	9%	7%
Lacked experience	Actor(s)’ lack of experience or knowledge led to the failure. For example, an inexperienced manager who was placed in charge of a large project.	42%	40%
Used inadequate justification	Actor(s) in the organization used inadequate justification for a decision.	42%	47%
Subjected to inadequate reviews	Actor(s) in the organization did not review documentation or other work sufficiently to capture errors and deficiencies.	12%	17%
Kept poor records	Actor(s) in the organization kept poor records of a process, such as maintenance.	21%	27%
Failed to form a contingency plan	Actor(s) in the organization failed to form a contingency plan to implement if an unplanned event occurred.	27%	20%
Inadequately communicated	Actor(s) in the organization failed to communicate with each other such that personnel were confused with the information they were given, had to “fill in the gaps” in the information they were given, or not notified about important information at all.	33%	23%
Conducted poor requirements engineering	Actor(s) in the organization did not lay out the needs, attributes, capabilities, characteristics, or qualities of the system well.	52%	40%
Failed to consider human factor	Actor(s) in the organization failed to consider a human factor in system development. This causal action describes, for example, failing to consider human factors in specifying procedures or physical design.	30%	47%
Failed to inspect	Actor(s) in the organization failed to inspect a crucial component.	15%	37%
Violated regulations	Actor(s) in the organization violated a regulation pertaining to the system.	6%	33%
Managed risk poorly	Actor(s) in the organization failed to identify, assess, formulate, or implement a proper mitigation measure.	48%	77%
Subjected to inadequate testing	One or more actors in the organization subjected a component or subsystem to inadequate testing. This causal action captures inadequate tests as well as adequate tests performed inadequately.	45%	17%
Violated procedures	Actor(s) in the organization violated a procedure pertaining to the system, such as a maintenance or operation procedure.	21%	53%
Did not allow aspect to stabilize	Actor(s) in the organization did not allow a system aspect like personnel, design, or requirements to stabilize before moving forward with the project.	45%	7%
Did not learn from failure	Actor(s) in the organization did not take past failures into account and a similar problem occurred.	9%	50%
Conducted maintenance poorly	Actor(s) in the organization failed to perform maintenance on a component or subsystem.	3%	47%
Created inadequate procedures	Actor(s) in the organization developed a deficient procedure, for instance maintenance, manufacturing, or emergency procedures.	18%	63%
Enforced inadequate regulations	A regulator (e.g., the FAA) enforced deficient regulations. This causal action captures writing deficient regulations as well as implementing regulations poorly.	3%	50%
Failed to train	Actor(s) in the organization failed to train other actors in the organization, such as operations personnel or maintenance personnel.	12%	63%

The accidents and other project failures in our data set share many causes. Which causes are most often reported in accidents and project failures? Are some causes reported more in accidents than in project failures, and vice versa? To answer these questions, we define a *presence measure* that answers the question: “How often does a particular cause appear across the failure samples?” The presence measure for *cause*_*i*_ is given by:

$$presence(cause_{i})=\frac{\sum_{k=1}^{k=N}TRUE(cause_{i}|failure_{k})}{N}$$ (1)

Where *failuree*_*k*_ is the *k*^*th*^ accident or project failure and *N* is the number of accidents or project failures. For example, “failed to train” occurred at least once in 19 of the 30 accidents, so its accident presence is 63%. This cause occurred at least once in 4 of the 33 project failures, thus its presence in project failures is 12%. The presence measure is binary within failures, i.e., it does not assign greater weights to causes that appear multiple times within a particular failure. Thus, any double counting of causes within a failure (e.g., a cause that appeared in two different report sections) does not affect the presence. Table 4 shows the presences and definitions of the 23 causes, ordered from most to least similar frequencies.

Previously in this section we showed examples of causes that appear similar between project failures and accidents. Table 4 shows that many causes have similar presence between project failures and accidents, but others have quite different presences. In [38] we discussed in detail where and why causes are similar and different between accidents and project failures, and here we provide a brief summary of that discussion. The higher presence of some causes is likely an artifact of accident investigations generally being more detailed and thorough than project failure investigations. For example, we found far fewer instances of *inadequate procedures* in project failures than accidents. For procedures specifically, the systems that experienced accidents likely had procedures that were more clearly defined than those for project failures because procedures are explicitly required for system operation (not necessarily so for project development). In the Alaska Airlines flight 261 crash, when the horizontal stabilizer did not respond properly, the pilot attempted different control configurations until the faulty jackscrew completely gave way and the aircraft nose-dived into the ocean. The NTSB criticized the emergency procedures, stating: “Without clearer guidance to flight crews regarding which actions are appropriate and which are inappropriate in the event of an inoperative or malfunctioning flight control system, pilots may experiment with improvised troubleshooting measures that could inadvertently worsen the condition of a controllable airplane” [40, p. 140].

Some differences in cause presence may indicate actual differences between the types of failures. Notably, many of the project failures we studied occurred before the systems had matured through their design cycles and therefore had no opportunity to perform maintenance. Thus accidents had more instances of the cause *conducted maintenance poorly*. For instance, in the Three Mile Island nuclear accident, “[r]eview of equipment history for the 6 months prior to the accident showed that a number of equipment items that figured in the accident had had a poor maintenance history without adequate corrective action” [41, p. 47]. The single instance of this cause in project failures is in the Hubble spacecraft mirror flaw, in which the equipment used to manufacture the mirror (and responsible for the flaw) had been poorly maintained [42].

Since our sample is relatively small and was not selected randomly, we cannot definitively (and with statistical certainty) conclude that in project failures actors *failed to train* exactly twice as frequently as actors *violated regulations*. However, it is reasonable to conclude that actors in project failures *fail[ing] to supervise* is a more pervasive and visible problem than actors *fail[ing] to inspect*. Using this mindset, we recommend that practitioners looking for what problems may lead to project failures over accidents look for the causes with the highest frequencies from Table 4 for that failure (e.g. look for weaknesses in supervision prior to looking for weaknesses in maintenance).

### Study bias investigation

To determine whether our study suffered from strong indicators of bias, we enlisted an associate to perform the same extraction process on a few of the project failures we studied so we could perform an inter-rater agreement calculation on the result. We determined the presence (see Eq 1) of each cause from the associate’s process and compared this result to the presence we determined from our coding process to calculate the percent agreement. Table 5 shows the results of our analysis. The average inter-rater agreement was 82%, which indicates “very good” high inter-rater agreement [43], and is a good indication that our process is free from rater bias.

**Table 5:** Inter-rater agreement results.

Project Failure	Percent Agreement
Xbox 360 gaming console	78%
Iridium satellite phone	78%
Boston Big Dig infrastructure project	83%
Navy Seawolf submarine	91%
F-22 Raptor	96%
Future Imagery Architecture Satellite Project	70%
Hubble Space Telescope Mirror Flaw	78%
**Average**	**82%**

### Recommendation extraction and analysis

Project failure reports rarely contain recommendations. Only one of the project failures we studied contained recommendations (the Drug Enforcement Administration (D.E.A.) plane [44]), and these recommendations do not address the underlying problems that led to the failed acquisition. In contrast, most large accident investigations include extensive recommendations on how to prevent future accidents. Since we have found that accidents and project failures share many causes, recommendations from accident investigations are potentially also applicable to project failure prevention.

**Fig 4:**
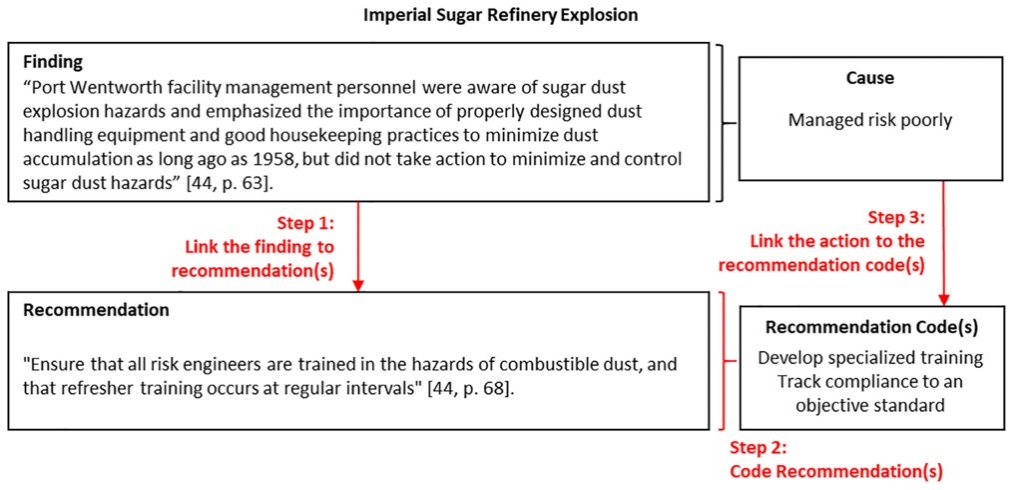
Recommendation coding and linking diagram (Imperial Sugar Refinery accident).

Fig 4 describes our approach to coding and analyzing the recommendations from accident reports, using excerpts from the Imperial Sugar Refinery Accident report [45]. First, we linked the accident report findings to the corresponding recommendations. Some accident reports explicitly link recommendations to specific findings (e.g., the Space Shuttle Columbia accident report [46]), but most of the reports do not. For example, NTSB reports have a section labeled “findings” followed by a section labeled “recommendations”, but in general there is no explicit link to the recommendations from the findings. One of the reports did not make any recommendations at all (the Bhopal accident [47]) and others made only a few recommendations, often addressing only a subset of the findings.

We used a similar approach to the cause coding to code the recommendations. Some findings had multiple recommendations that spanned many ideas, so a single cause could have more than one recommendation, and hence potentially more than one recommendation code. In Fig 4, we connected the finding to a single recommendation, which we described using two recommendation codes because it contains two distinct ideas. In total, we identified 16 recommendation codes, as shown in Table 6.

**Table 6:** Recommendation code definitions [14].

Recommendation	Definition
Conduct random and independent evaluations	Perform an evaluation like an inspection or audit on a component, organization, or person, and do it randomly, often, and by an independent organization or party.
Develop a comprehensive and rigorous test	Develop a test that includes all possible regimes, equipment, and situations, and is stricter than what is minimally necessary (e.g. to a certain factor of safety).
Develop specialized training	Develop training to teach, reiterate, or reinforce a specific aspect related to the failure.
Establish a program or service	Establish a program to aid a process, such as a record-keeping program.
Establish an independent and transparent supervisory agency	Establish an agency that acts as a watchdog for an aspect of the failure.
Establish more checks in the system	Put more checks in the system, for example a supervisor’s signoff, such that work cannot continue without conducting the check.
Give supervisor more capacity for oversight	Provide supervisors with the power to enforce the rules to which systems are required to adhere.
Identify weak areas	Assess what aspects of the system may be neglected.
Improve efficiency in critical tasks	Improve how a task is done, for example by eliminating steps in a procedure, providing better equipment, or making software assistance to operators more logical.
Increase resources	Provide more aspects like people, money, or equipment, to an aspect of the system.
Involve stakeholders in decision-making	Involve more stakeholders to provide additional points of view that were previously lacking.
Keep up with current technologies	Improve technological aspects of the system like outdated computer systems, or emergency systems.
Make instructions more clear	Improve instructional aspects of the system, such as procedures, job descriptions, employee roles, or any other type of instruction to be clearer.
Make regulations more strict	Improve regulations to make the standards to which the system is held to be more stringent.
Review decision-making logic	Instead of incrementally making small changes to a system, rather, for example, change how aspects of the system are addressed or review the system from a high-level perspective.
Track compliance to an objective standard	Hold system activities to applicable standards, such as ensuring drawings follow a template or having every employee complete the same training.

**Fig 5:**
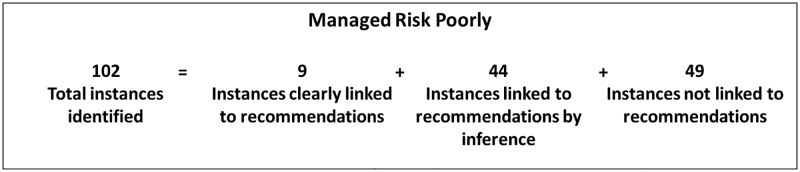
Recommendation code distribution for *managed risk poorly*.

Last, we linked the causes from the actor-causal action-object codes to the recommendation codes. We linked only those recommendations that we could reasonably infer corresponded to the causes we identified. Fig 5 displays the recommendation code distribution for *managed risk poorly*. Overall, we did not find recommendations for 30% of the accident causes.

This cause-recommendation linking effort has shown that the recommendations made in accident reports are not without flaws. First, the effort needed to link causes and recommendations (those that are linked by inference) highlights a lack of clarity in accident reports. Second, we were not able to link many causes to recommendations, which indicates that there are problems the investigators found that they (1) did not have the resources to make a recommendation for, (2) did not know how to solve, or (3) did not think was critical enough to improve upon. Nevertheless, the recommendations made in accident reports are likely more useful than those we found in the project failure literature because they provide more specific, actionable guidance.

## Cause networks and the cause-recommendation network

We have identified over 1,200 specific examples of failure causes and 800 specific examples of remedial actions. Here, we develop a graphical network to facilitate navigation of the results.

### Cause network

The cause network is based on the cause presence and the probabilities of finding pairs of causes in a given accident or project failure. Table 7 shows the intersectional probabilities *P*(*cause*_*i*_ ∩ *cause*_*j*_) for “failed to consider human factor” (*cause*_*i*_) and all the other causes for both accidents and project failures. For example, *failed to supervise* occurred together with *failed to consider human factor* in 21% of project failures, and 37% of accidents.

**Table 7:** Intersectional probability of *failed to consider human factor* with the other causes.

*cause*_*j*_	*P*(*failed to consider human factor* ∩ *cause*_*j*_)	
	Project Failure	Accident
Failed to supervise	21%	37%
Failed to provide resources	18%	23%
Failed to consider design aspect	27%	37%
Lost tacit knowledge when employee departed	6%	3%
Lacked experience	15%	20%
Used inadequate justification	6%	23%
Subjected to inadequate reviews	3%	10%
Kept poor records	6%	13%
Failed to form a contingency plan	12%	10%
Inadequately communicated	18%	10%
Conducted poor requirements engineering	15%	13%
Failed to inspect	9%	17%
Violated regulations	3%	20%
Managed risk poorly	18%	30%
Subjected to inadequate testing	12%	7%
Violated procedures	6%	23%
Did not allow aspect to stabilize	15%	3%
Did not learn from failure	3%	27%
Conducted maintenance poorly	0%	27%
Created inadequate procedures	6%	30%
Enforced inadequate regulations	0%	27%
Failed to train	0%	40%

**Fig 6:**
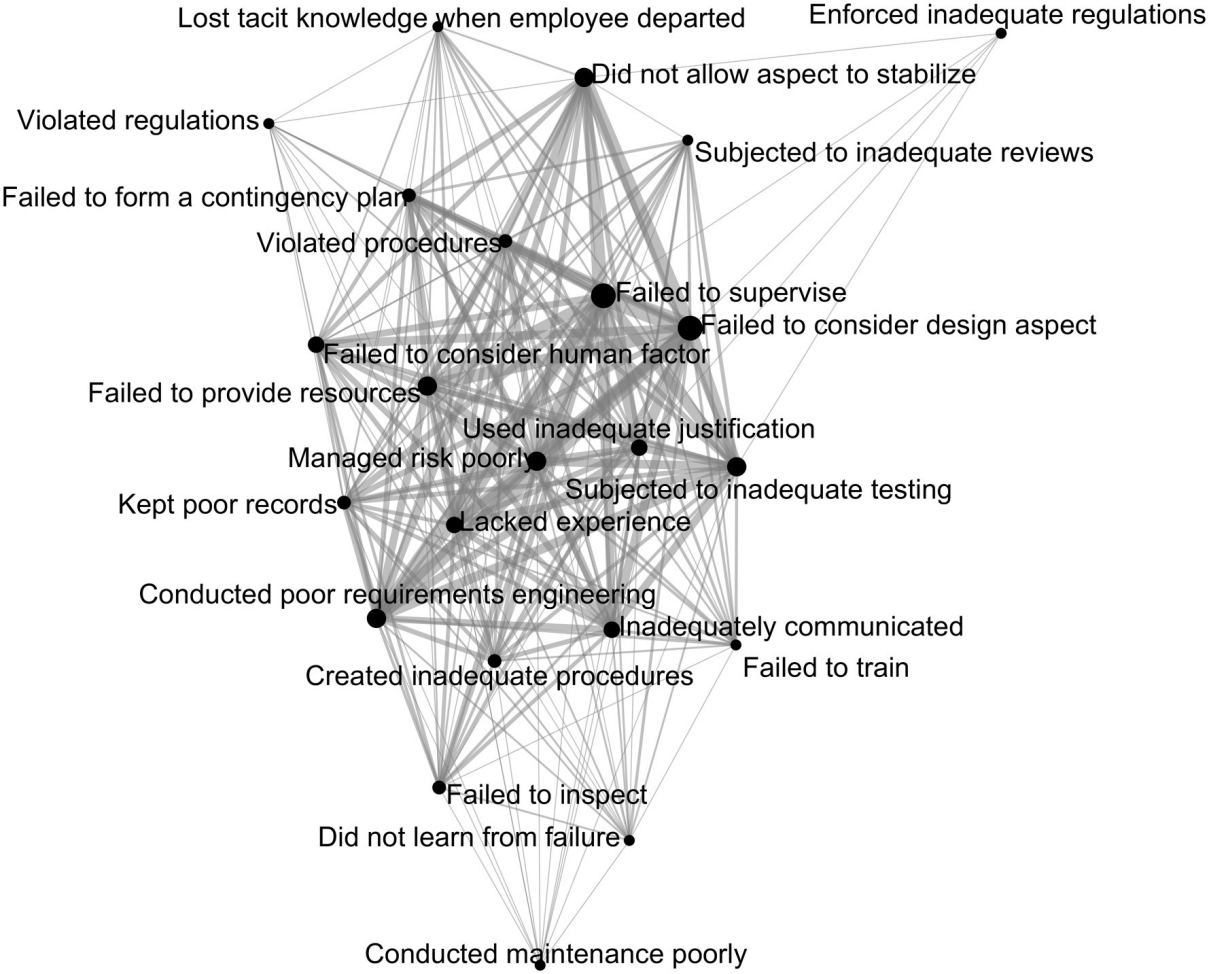
Project failure cause intersection likelihood.

**Fig 7:**
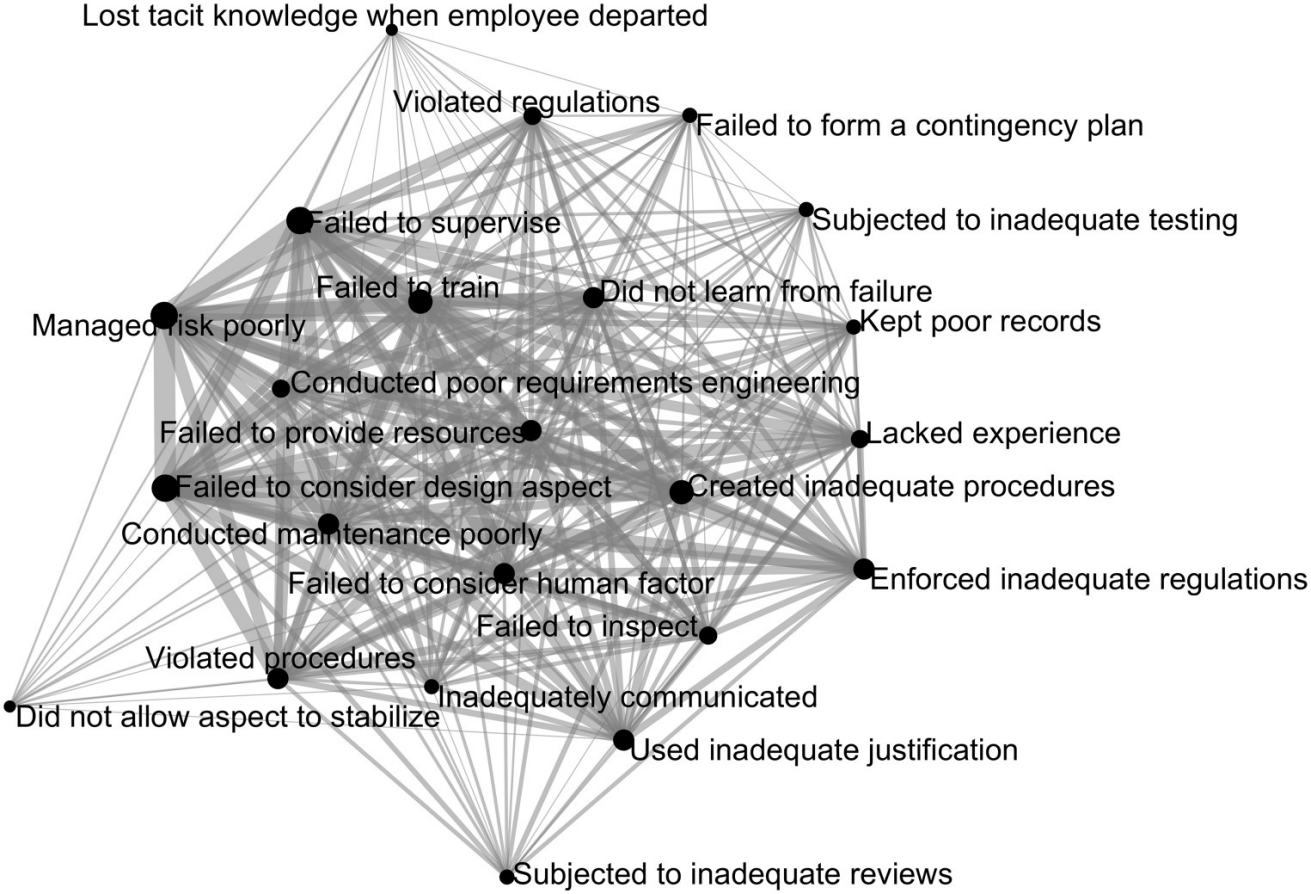
Accident cause intersection likelihood.

We plotted the intersectional probabilities of causes for accidents and for project failures as undirected graphs, as shown in Figs 6 and 7. The nodes represent the causes, and the links represent the cause intersectional probabilities. Heavy links indicate high intersectional probabilities, thin links the opposite. Large nodes indicate a high cause presence, small nodes the opposite. Linked nodes appear closer to each other, and unlinked nodes appear further from each other. In project failures (Fig 6) the eight causes with low presence (<20%), such as *enforced inadequate regulations*, are all outlying nodes with thin connections. Similarly, the five causes with low presence in accidents (Fig 7), such as *did not allow aspect to stabilize*, are all outlying nodes with thin connections. The two causes with high presence (>70%) in project failures (*failed to consider design aspect* and *failed to supervise*) are both internal nodes in with many thick connections. Similarly, the three causes with high presence in accidents, such as *managed risk poorly*, are also internal nodes with many thick connections. Fig 6 has more outlying nodes, with thinner connections on average than Fig 7. The causes in project failures generally have lower presence values than causes in accidents, which means there are fewer opportunities to be connected to the other causes.

### Cause-recommendation network

**Fig 8:**
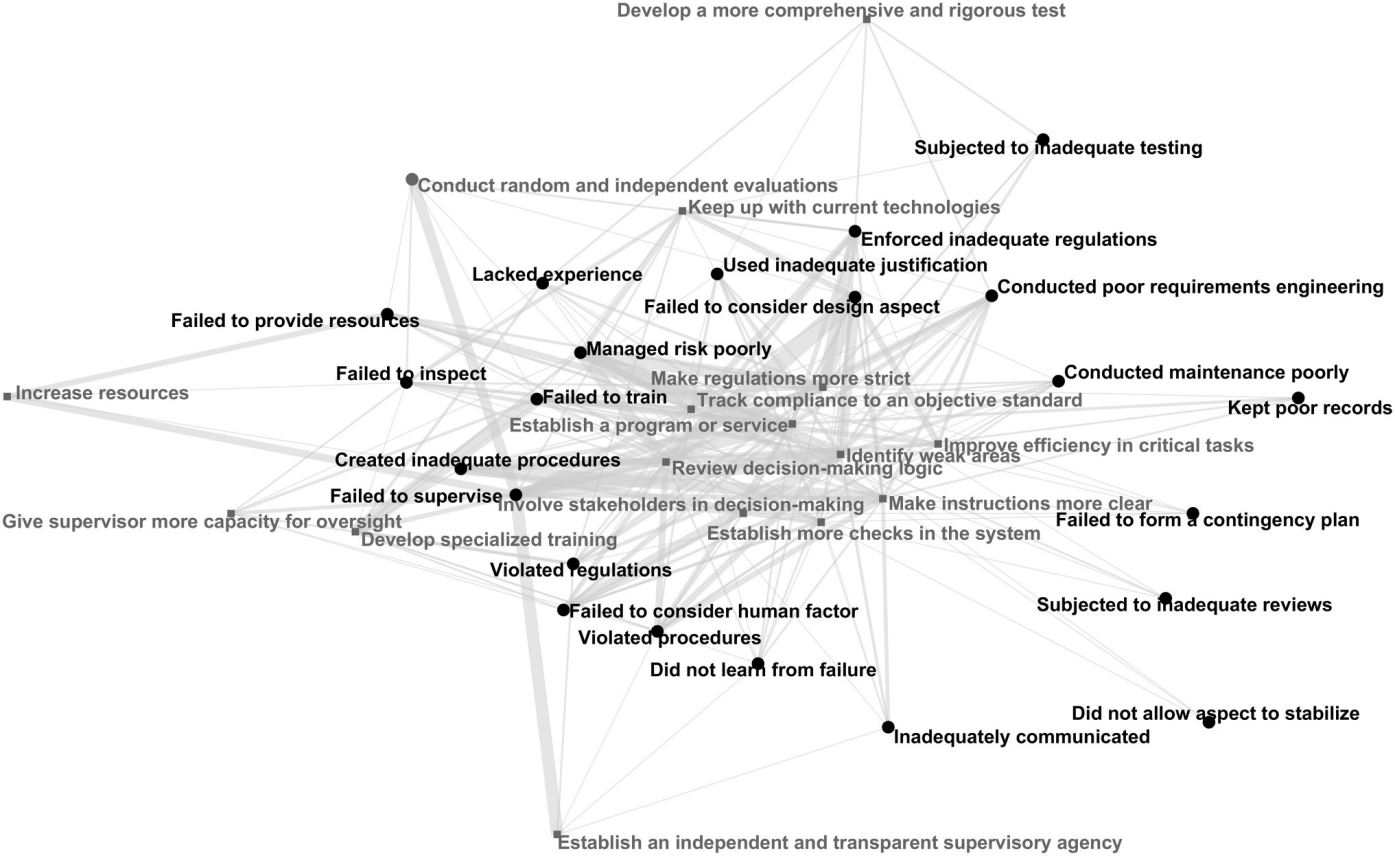
Cause-recommendation network.

Next, we built a cause-recommendation network using the links we identified between the causes and the recommendation codes. In Fig 8, the black nodes are causes, and the gray nodes are recommendations. For clarity, we have omitted the cause-cause links. Like the cause networks, nodes with many connections repel nodes with few connections. Thin links indicate that the cause and recommendation were connected only one or two times; heavy links the opposite, with the thickest line indicating 49 connections between *managed risk poorly* and *no recommendation* (see Fig 5). Some causes only have a few recommendations; this situation occurs when causes are quite specific and also have quite specific recommendations. For example, a frequent recommendation for *subjected to inadequate testing* is *develop a more comprehensive and rigorous test* (that is, a frequently suggested solution to inadequate testing is adequate testing!). Other causes are more ambiguous and are thus covered by a wider range of recommendations. Such causes include *failed to supervise*, which is covered by recommendations like *conduct random and independent evaluations* and *develop specialized training*.

## Application of the network

In the Introduction, we discussed how suggestions for improvement are often either so general they are essentially platitudes (“put your best people on the job”), or highly specific to particular contexts (e.g., “replace the faulty burst valve”). In contrast, our study straddles both of these approaches to not only provide practitioners general language to help them categorize their problems but also provide specific examples of each of these general problems in a wide variety of industries and contexts. Subsequently, many of these specific problems have expert-provided recommendations that practitioners may use as inspiration for solving their own problems. Here, we demonstrate two aspects of how the information in the cause-recommendation network can be used to identify useful and informative guidance.

### Identifying and understanding potential causes

An organization that suspects it may have problems can use the network to identify the most frequent causes. Our analysis of project failure and accident causes showed where most of the problems are likely to be found for either type of event. In the cause extraction section, we suggested that an organization looking for what problems may lead to failures look for the causes with the highest frequencies from Table 4 for that failure type.

The most frequent cause in both accidents and project failures is *failed to consider design aspect* (Table 4). To help illustrate this and the other causes, the network also provides over 900 “back stories” of how each cause has appeared in accidents and project failures. Table 8 shows examples of these back stories from both accidents and project failures for *failed to consider design aspect*.

**Table 8:** Back stories for *failed to consider design aspect*.

Failure	Cause Back Story
Upper Big Branch Mine explosion [48]	The Upper Big Branch Mine was a coal mine in West Virginia that suffered an explosion that killed 29 miners. Coal mines require constant “rock dusting” to keep coal dust levels down to prevent explosive atmospheres from forming within the mine. Among other causes, the mine was so large that workers conducting rock dusting had to make many trips to reload material to rock dust the entire mine.
	**Design aspect not considered:** A chute-like delivery system to the center of the working area of the mine would have made rock dusting easier.
ValuJet flight 592 crash [49]	Contractors working for ValuJet Airlines were refurbishing an aircraft and removed its expired chemical oxygen generators, used to supply oxygen to passengers in situations when a plane suffers a decompression during flight. The contractors improperly packaged and labeled the generators as empty rather than expired. Eventually the expired, but not empty, generators were shipped on flight 592. During takeoff, a fire started in the cargo hold, and would have burned itself out had the (now damaged) generators not supplied the fire with oxygen. The plane was eventually overwhelmed by the fire and crashed. The passengers and crew were all killed on impact. The NTSB report also noted that even if the aircraft had managed to land, the passengers might have been injured or killed by toxic air.
	**Design aspect not considered:** The emergency oxygen masks deployed during in-flight emergencies do not separate cabin air, which could be toxic in the event of a fire, from the oxygen flow.
Westray Mine collapse [39]	The Westray Mine was a coal mine in Nova Scotia that had a history of problems because the mine’s management frequently took shortcuts to improve production at the cost of safety.
	**Design aspect not considered:** the ventilation system in the mine was designed in a haphazard way; for example, the fans were placed in locations within the mine that were not conducive for the air flow. Thus, the ventilation system allowed methane gas and coal dust to build up, eventually causing the mine to collapse.
Iridium satellite phone cancellation [50]	The Iridium satellite phone was a phone that could connect a call anywhere on Earth at a time when cell phone coverage was unreliable. The phone did not sell well, and the founding company declared bankruptcy, although the satellite system remains operational.
	**Design aspect not considered:** Designers did not properly consider their customers’ needs. The phone was extremely expensive, calls could only be made outside (within line-of-sight of the satellite network), the phone was difficult to use and required special training, a special cartridge was required to make conventional mobile network calls, and the phone itself was large, weighing over 1 lb.
Seawolf Navy Submarine delays and cost overrun [51]	The Seawolf Navy submarine was delayed and over-budget.
	**Design aspect not considered:** Two contractors who had originally competed to win the contract were commissioned to design and build the aft and forward sections of the submarine separately. This decision underestimated the immense coordination and cooperation that would be required between the contractors, as well as the extensive design and construction rework the program eventually needed.
F-35 Lightning II delays and cost overrun [31]	The F-35 Lightning II is currently delayed and over-budget.
	**Design aspect not considered:** The aircraft is intended to be one-size-fits-all for the United States Navy, Air Force, and Marines, which means that a single design, with slight modifications, is meant to meet the needs of all three customers. This common platform decision did not fully consider the challenges and compromises involved in trying to meet divergent needs. In addition, development on all three variants is delayed whenever a common part fails.

These examples show the pitfalls of major design decisions, such as having two (formerly competing) contractors build separate ends of a large system while neglecting coordination effort or how delayed common parts in the development of a program can snowball to cause large-scale delays. A practitioner who is interested in the ramifications of issues like failing to consider certain aspects of design could peruse these examples.

### Identifying and understanding potential recommendations

**Fig 9:**
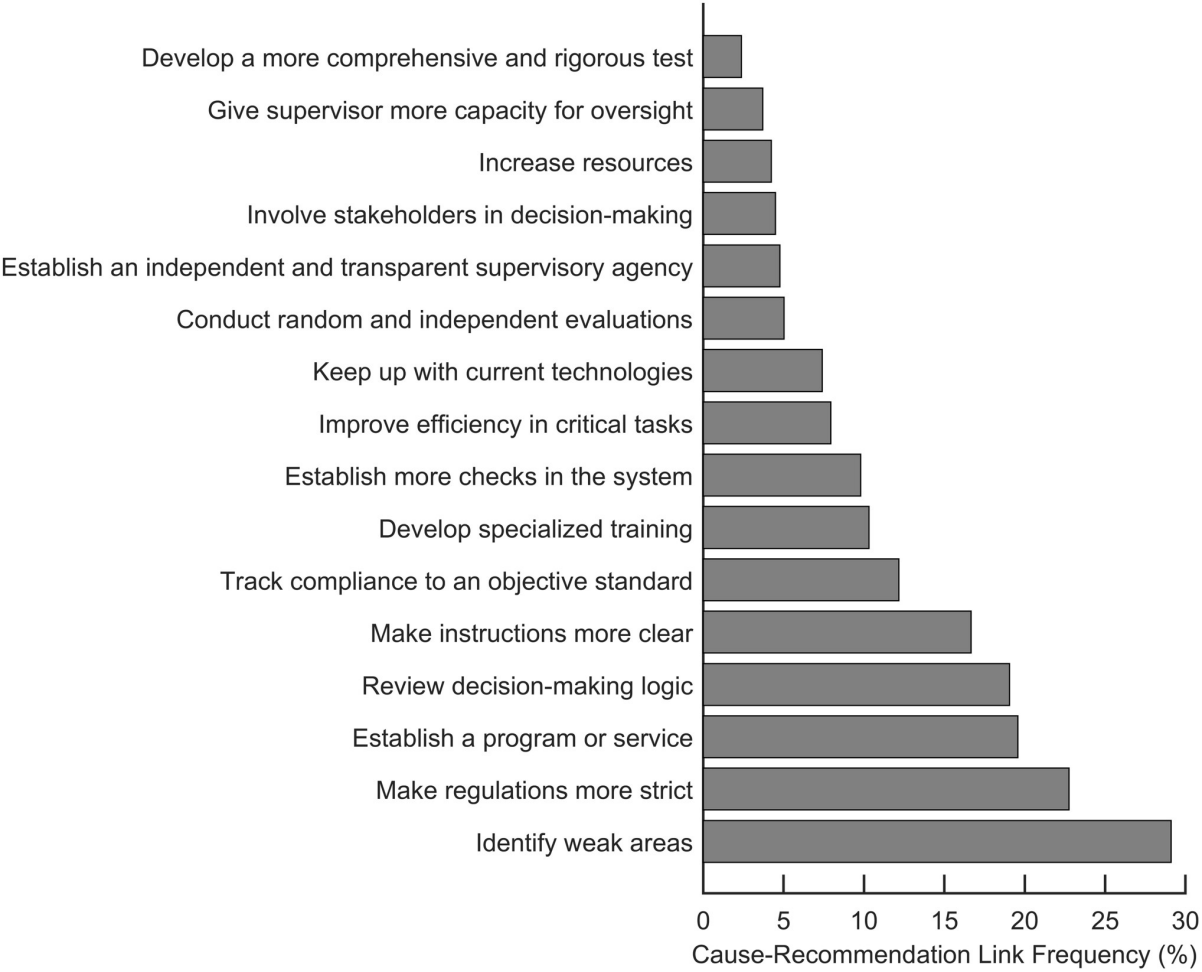
Recommendations ranked by cause-recommendation link frequency in accidents.

Practitioners may find it useful to see what general improvements accident investigators most often recommended to make cost-effective and efficient resourcing decisions on their project. Fig 9 shows the 16 recommendations, ranked by the percentage of accident causes connected to each one. The percentages do not add up to 100% because many causes are linked to more than one recommendation code (see Fig 4) and some causes are not linked to any recommendations. For example, *make instructions more clear* accompanied 17% of the causes in accidents that had recommendations. An organization seeking to make general improvements without prior knowledge of problems should start by following the recommendation codes with the highest percentages. These recommendations are most likely, based on our dataset, to be applicable in any given organization.

In Fig 9, It is not surprising that *identify weak areas* was most often recommended—it is hard to imagine a scenario in which identifying weak areas is not a good idea! Similarly, many of the other recommendations also appear self-evident, but may be hard to translate into concrete context-specific terms. To help address this problem, the cause-recommendation network provides 600 back stories of the recommendations and the problems that led to the recommendations. For example, Table 9 shows examples of why and how investigators made the recommendation *identify weak areas*, which appears in 25 out of 30 accident investigations and is linked to 29% of accident causes.

**Table 9:** Examples of source accidents for recommendation code *identify weak areas*.

Code: Identify weak areas (29%): Assess what aspect of the system is potentially neglected.		
Cause	Accident	Recommendation
Created inadequate procedures	Swissair Flight 111 Crash [52]	A fire started on the aircraft while it was flying, and because it propagated in unoccupied parts of the aircraft, it went unnoticed and eventually brought the plane down. Investigators found that the in-flight entertainment system was improperly installed on the aircraft, and as a result wires from the system chafed against metal components in the attic area of the aircraft. To identify discrepancies for all aircraft of this model in service, the investigators recommended that the FAA require an inspection for wiring discrepancies, such as chafed or cracked wire insulation.
Subjected to inadequate testing	Deepwater Horizon oilrig blowout and fire [29]	The Deepwater Horizon oilrig was plugging a new oil well in a standard procedure. The well’s plug burst and the ensuing oil spurting from the well caught on fire and destroyed the rig. When the crew performed a test to determine the plug’s integrity, the test results indicated that the plug was not secure, but instead of investigating the results, the crew performed a different test and mistakenly concluded the plug was secure. The investigators recommended that the regulator require oilrig operators seeking design approval to demonstrate that well components are equipped with sensors or other tools to obtain accurate diagnostic information on the status of the well.
Failed to consider design aspect	Buncefield oil fire [53]	The Buncefield oil storage depot was filling a storage tank with oil when a gauge designed to detect when the oil reached a high point failed. There was no alarm and the receiving site could not halt the flowing oil. The tank overfilled and a spark lit the spewing oil on fire, causing an explosion. Operators relied on a retaining wall around the tank as a backup system to ensure that liquids would not be released to the environment. However, this containment method failed and oil and firefighting liquids flowed off site and entered the groundwater. The investigators recommended that operators of oil storage sites evaluate the siting and protection capabilities of emergency response measures at their facilities for potential weaknesses.
Created inadequate procedures	Colgan 3407 crash [54]	Colgan Air flight 3407 was on approach to Buffalo in icing conditions, and the pilot had the aircraft on autopilot, which made it more difficult for him or the co-pilot to realize that the wings were icing. Neither the pilot nor the co-pilot responded appropriately to stall warnings and were not able to recover the aircraft from the stall, leading to the aircraft crashing. Pilot fatigue played a role in the pilots responding inappropriately, and at the time Colgan did not provide any information to its pilots about fatigue prevention. The investigators recommended that airline operators address fatigue risks associated with pilot commutes, including identifying pilots who commute and providing guidance to mitigate fatigue risks.
Failed to consider human factor	Texas City refinery explosion [55]	The Texas City Refinery was a plant that refined oil into unleaded petrol. As part of this process, the plant used a raffinate splitter that separated as much as 45,000 barrels of the fluid into lighter and heavier hydrocarbon components using a tall tower. A combination of factors led to the relief system for the tower overfilling and raffinate fluid spilling out, creating a flammable vapor cloud that was eventually ignited by a nearby pick-up truck engine a worker had left running. In particular, the control board display for the process did not provide adequate information to the operator, such as the imbalance of the flow of hydrocarbons. The CSB recommended that the plant evaluate its process units to ensure that critical process equipment is safely designed, such as by having effective instrumentation and control systems and by configuring control board displays to clearly indicate material balance.

If an organization has identified a particular problem behavior, it can use the cause-recommendation network to identify the most appropriate recommendations for addressing that behavior. For example, suppose an organization discovers that it did not adequately supervise a project. Table 10 shows the associated recommendations for *failed to supervise*, as well as the relative ranking of each recommendation, based on how often we connected them to *failed to supervise*, described in percentage as well as raw count. Thus, for example, *identify weak areas* was recommended 16 times in response to *failed to supervise*, which we identified a total of 117 times in our accidents and project failures. Thus its percentage is 16/17 ≈ 14%.

**Table 10:** Recommendations for causal action *failed to supervise* [26].

Recommendation Code	%	#
Identify weak areas	14%	16
No recommendation code	14%	16
Establish an independent and transparent supervisory agency	11%	13
Establish a program or service	10%	12
Conduct random and independent evaluations	9%	11
Make regulations more strict	8%	9
Increase resources	7%	8
Review decision-making logic	5%	6
Track compliance to an objective standard	5%	6
Develop specialized training	4%	5
Make instructions more clear	4%	5
Give supervisor more capacity for oversight	3%	4
Involve stakeholders in decision-making	2%	2
Keep up with current technologies	2%	2
Establish more checks in the system	1%	1
Improve efficiency in critical tasks	1%	1
Develop a more comprehensive and rigorous test	0%	0
**Total**	**100%**	**138**

The network also allows users to sort by other categories, such as industry type—a user could, for instance, see all causes related to government acquisitions or aircraft crashes.

Our work is currently available in an interactive web-based platform, where the user is able to click on a certain cause, see what other causes are related to that selected cause, and then see recommendations related to that set of causes, available at: https://engineering.purdue.edu/VRSS/research/force-graph/index_html. For details on how we constructed this interactive version of the network and how we propose practitioners use the network to identify problems and potential solutions in their own organizations, see [14].

To see our preliminary results on using this network with novice and expert systems engineers to determine whether this tool is useful for forming remediation measures for problems on projects, refer to [38].

The source data for this research is available on the Purdue University Research Repository: https://purr.purdue.edu/publications/2859.

## Conclusion and future work

We identified a set of 30 accidents and 33 project failures, spanning a wide range of industries. Next, we modified Leveson’s STAMP model and used it to methodically extract and analyze their causes. We found 23 different failure causes, most of which appear in both accidents and other project failures, suggesting that accidents and project failures do happen in similar was. We also identified 16 different recommended remedial actions. We link these causes and recommendations in a cause-recommendation network, and associate over 900 specific examples of how these causes manifested in failures, and over 600 specific examples of the associated recommended remedial actions, with each cause or recommendation.

The limitations of this study are such: first with identifying project failures to study. As Judgev & Müller [56] state in their paper on understanding project success: “Trying to pin down what success means in the project context is akin to gaining consensus from a group of people on the definition of ‘good art’.” Not only is project success difficult to define, but project failure is also not one-minus the definition of project success. Readers may disagree with the way in which we defined project failures (e.g. we classified unmanned space mission failures as project failures, but we classified the Space Shuttle disasters in which the crews were killed as accidents), but this distinction has no material effect on our results and our results are potentially useful for any project experiencing problems, no matter the distinction. Second, studying a set of previously-reported project failures and accidents is inherently subject to bias from the investigators. These biases are inherent to any approach based on studying investigation reports. We discuss these potential biases at length in [34]. Third, the extraction and coding process is subject to bias by the coders. Different coders may identify more or fewer causes or recommendations in a given report, and different coders may assign a given finding or recommendation to different codes. Since we provide in the network both the original sources and the paraphrased “stories” behind each instance of each code, the impact of the code creation and allocation process is minimal.

In this paper, we focused on the causes. In future work, we will expand the network by incorporating other aspects from our analysis, for instance (1) The actors involved in each cause, (2) The types of objects involved in the causes and the difference between project failures and accidents (e.g. what types of testing was involved), or (3) When in the design cycle the cause occurs. Companies experiencing problems during project development may use the cause-recommendation network as a guide to analyze any issues they have found, identify other potential related issues, and then use the recommendation codes to reduce the likelihood of failure.

We developed a specialized coding scheme to compare the causes of systems engineering related accidents and project failures. There are also other coding schemes, both more general and more specific, such as the HFACS accident causation hierarchy. Part of our future work may include mapping our coding scheme to other methods to analyze the differences in the coding schemes and determine whether different patterns emerge.

Adding findings to the network is easy, but extracting and coding them requires significant effort. Machine learning methods may provide an automated way of adding failures to our cause-recommendation network [57] [58] [59].

Finally, in related work we are using game theoretic approaches to explore the underlying reasons behind the causes we identified here [60].
